# Altered Brain Structure and Spontaneous Functional Activity in Children With Concomitant Strabismus

**DOI:** 10.3389/fnhum.2021.777762

**Published:** 2021-11-15

**Authors:** Xiaohui Yin, Lingjun Chen, Mingyue Ma, Hong Zhang, Ming Gao, Xiaoping Wu, Yongqiang Li

**Affiliations:** ^1^Department of Radiology, The Affiliated Xi’an Central Hospital of Xi’an Jiaotong University, Xi’an, China; ^2^Department of Radiology, Gaoling District Hospital, Xi’an, China; ^3^Department of CT and MRI, Weinan Hospital of Traditional Chinese Medicine, Weinan, China

**Keywords:** concomitant strabismus, cortical thickness, ALFF, structural MRI, resting-state fMRI

## Abstract

Strabismus occurs in about 2% of children and may result in amblyopia or lazy eyes and loss of depth perception. However, whether/how long-term strabismus shapes the brain structure and functions in children with concomitant strabismus (CS) is still unclear. In this study, a total of 26 patients with CS and 28 age-, sex-, and education-matched healthy controls (HCs) underwent structural and resting-state functional magnetic resonance imaging examination. The cortical thickness and amplitude of low-frequency fluctuation (ALFF) were calculated to assess the structural and functional plasticity in children with CS. Compared with HCs group, patients with CS showed increased cortical thickness in the precentral gyrus and angular gyrus while decreased cortical thickness in the left intraparietal sulcus, parieto-occipital sulcus, superior and middle temporal gyrus, right ventral premotor cortex, anterior insula, orbitofrontal cortex, and paracentral lobule. Meanwhile, CS patients exhibited increased ALFF in the prefrontal cortex and superior temporal gyrus, and decreased ALFF in the caudate and hippocampus. These results show that children with CS have abnormal structure and function in brain regions subserving eye movement, controls, and high-order cognitive functions. Our findings revealed the structural and functional abnormalities induced by CS and may provide new insight into the underlying neural mechanisms for CS.

## Introduction

Concomitant strabismus (CS) is the most common type of strabismus and is characterized by an equal angle of ocular misalignment in all fields of gaze, regardless of which eye is used for fixation ocular motility disorders (Robaei et al., 2006). CS develops most commonly in early childhood and presents with abnormal eye position and poor stereopsis (Oystreck and Lyons, 2012). Alternatively, children with CS often suffer from several psychosocial and emotional consequences, for example, negative social bias, increased social anxiety, and poor interpersonal relationship (Archer et al., 2005). Nevertheless, beyond the clinical data, the pathological mechanisms underlying CS remain unclear. Accordingly, it is urgently needed to deepen the current understanding of the etiology of CS, which may provide new clues of the planning of the surgery.

Magnetic resonance imaging (MRI) techniques have developed rapidly to provide a non-invasive neuroimaging method that can characterize the structural and functional changes in the brain (Singh et al., 2013; Pirnia et al., 2016; Wang et al., 2017, 2019; Xu et al., 2019b). Using structural MRI, decreased gray matter volume (GMV) and white matter volume (WMV) in the middle temporal gyrus, cerebellum posterior lobe, posterior cingulate cortex, and premotor cortex were found in CS patients, and the decreased GMV and WMV were negatively correlated with duration (Ouyang et al., 2017). In addition, patients with CS also exhibited abnormal fractional anisotropy and mean diffusivity vales in the prefrontal cortex (PFC), superior temporal gyrus, globus pallidus/brainstem, precuneus, anterior cingulate, and cerebellum posterior lobe (Huang et al., 2016a). Measuring the spontaneous brain activity, resting-state functional MRI study reported abnormal amplitude of low-frequency fluctuation (ALFF) values in the medial PFC, angular gyrus, and cerebellum posterior lobe, and the changed ALFF values in these areas were correlated with the degree of depression in adults with CS (Tan et al., 2016). The increased regional homogeneity values in the inferior temporal cortex, fusiform gyrus, cerebellum anterior lobe, lingual gyrus, and cingulate gyrus were also reported in adults with CS (Huang et al., 2016b). Moreover, CS patients had increased functional connectivity between the posterior primary visual cortex and other oculomotor regions (Yan et al., 2019). These findings suggest significant brain abnormalities in CS, which may underlie the pathologic mechanisms of fusion defects and ocular motility disorders in patients with CS. However, the above mentioned studies were focused on adults with CS, whether/how the structure and function change in children with CS remains unclear.

In the present study, the cortical thickness and ALFF were calculated in order to measure the brain structure and intrinsic activity in children with CS. Based on previous literature, we hypothesized that children with CS would exhibit abnormal cortical thickness and ALFF in brain regions subserving eye movement, cognition, and emotion.

## Materials and Methods

### Participants

A total of 26 patients with CS (16 males and 12 females) were recruited from the affiliated Xi’an Central Hospital of Xi’an Jiaotong University. All patients were diagnosed by two experienced ophthalmologists according to the diagnostic criteria of the Chinese Medical Association. The inclusion criteria of CS were: (1) age with 8–15 years; (2) Naked or corrected visual acuity ≥0.8, and (3) children with no anisometropia, ocular and systemic organic diseases. The exclusion criteria were: (1) amblyopia, (2) patients with a history of previous ocular surgery, including intraocular and extraocular surgery, and (3) mental illness, brain trauma, and major neurological disorders. Twenty-eight healthy controls (HCs) who were closely matched in age, gender, and education level with patients with CS were recruited through advertisements. In all participants, the current severity of anxiety and depression was evaluated using the Hamilton Anxiety Scale (HAMA) and 17 items Hamilton Depression Rating Scale (HAMD), and cognitive function was assessed using the Montreal Cognitive Assessment scale (MoCA). The written informed consent was obtained from all participants before experimentation. This study was approved by the research ethics committee of the affiliated Xi’an Central Hospital of Xi’an Jiaotong University and is in accordance with the latest revision of the Declaration of Helsinki.

### MRI Acquisition

The data of all participants were collected using a Philips 3.0 T MRI scanner. Head movement and scanner noise were controlled using foam pads and headphones. Participants were instructed to keep their eyes closed, not think of anything, not fall asleep, and keep their head motionless during scanning. The T1 structural image was scanned using the following parameters: repetition time/echo time = 8.2/3.7 ms, inversion time = 1,100 ms, flip angle = 7°, acquisition matrix = 256 × 256, field of view = 256 × 256 mm^2^, voxel size = 1 × 1 × 1 mm^3^ and no gap. Resting-state fMRI data were obtained using an echo-planar imaging sequence with the following parameters: repetition time/echo time = 2,000/30 ms, matrix size = 64 × 64, field of view = 230 × 230 mm^2^, voxel size = 3.6 × 3.6 × 3.6 mm^3^, flip angle = 90°, 38 slices, gap = 0.6 mm, and 240 volumes.

### Structural MRI Preprocessing

The T1-weighted images were processed using the recon-all command in Freesurfer package 6.0.0 to generate the cortical surface and cortical thickness of the whole brain. The detailed pipeline has been described elsewhere (Dale and Sereno, 1993; Dale et al., 1999). For each subject, the preprocessing stream included motion correction, removal of non-brain tissue, transformation to Talairach space, segmentation of gray/white matter tissue, reconstructing the pial surface and surface of the gray/white junction, inflation of the folding surface plane, and topology correction (Fischl et al., 1999, 2002). Then, all surface data were visually inspected and inaccuracies were manually corrected. The thickness was defined as the shortest distance between the gray/white junction and the pial surfaces at each vertex across the cortical mantle. In order to arrive at the accurate matching of thickness measurement locations among participants, the cortical thickness maps were mapped to an average surface and were smoothed using a Gaussian kernel with a full width at half maximum (FWHM) of 10 mm prior to statistical analysis.

### fMRI Preprocessing

Image preprocessing was performed using the DPARSF toolbox^1^ based on SPM12^2^. The first 10 volumes were discarded before subsequent processing. The remaining 230 volumes were slice-timing and head motion correction. All participants were retained under the head motion criteria of translation <1.5 mm or rotation <1.5° in any direction. The data were then spatially normalized to the standard Montreal Neurological Institute (MNI) space with a voxel size of 2 × 2 × 2 mm^3^. Nuisance covariates included white matter, cerebrospinal fluid, and the Friston-24 parameters of head motion were regressed out from each voxel’s time course. The data were then smoothened with FWHM = 6 mm. The linear detrend and filtering (0.01–0.08 Hz) were performed to reduce the influence of low-frequency drift and high-frequency physiological noise. Subsequently, the filtered time series of each voxel was converted to the frequency domain using a Fast Fourier Transform and the power spectrum was then obtained. The averaged square root of the power spectrum was termed as ALFF. Lastly, the ALFF maps were standardized through division by the global mean of ALFF values.

### Statistical Analysis

The group differences of whole-brain cortical thickness between CS and HCs groups were conducted based on GLM models with MoCA, HAMD, and HAMA as covariates in Freesurfer, and the surface-based findings were corrected using family-wise error rate (FWE) with *p* < 0.05.

The ALFF distribution in the CS and HCs groups was obtained by averaging ALFF values at each voxel across subjects of each group. A two-sample *t-*test was performed to assess the group differences in ALFF between the CS and HCs groups with MoCA, HAMD, and HAMA as covariates. The result was multiple corrected using Gaussian random field (GRF) theory with cluster corrected *p* < 0.05 and a voxel height of *p* < 0.001.

### Correlation Analysis

Pearson correlation analyses were performed to evaluate the relationship between altered cortical thickness and ALFF of those identified regions and clinical performance, e.g., disease duration, HAMD, HAMA, and MoCA scores. The statistical significance was set at *p* < 0.05.

## Results

### Demographics and Clinical Characteristics

The demographic and clinical information of the HCs and patients with CS are presented in Table 1. There were no differences in age (*p* = 0.95), gender (*p* = 0.97), and years of education (*p* = 0.68) between two groups. However, the patients with CS showed higher HAMA (*p* = 0.026) and HAMD (*p* = 0.018) scores, whereas lower MoCA scores (*p* = 0.003) than HCs.

**Table 1:** Characteristics of demographic and clinical variables.

Variables	CS (*n* = 26)	HC (*n* = 28)	*P* value
Age (years)	10.77 ± 3.63	12.07 ± 3.69	0.95^a^
Gender (Male/Female)	15/11	16/12	0.97^b^
Education (years)	9.97 ± 4.18	9.66 ± 3.65	0.68^a^
Duration (years)	2.05 ± 1.22		
MoCA	21.27 ± 4.72	27.33 ± 2.63	0.003^a^
HAMA	5.72 ± 2.89	0.97 ± 0.86	0.026^a^
HAMD	6.04 ± 3.30	1.32 ± 0.14	0.018^a^

*Values are mean *±* standard deviation. MoCA, Montreal Cognitive Assessment scale; HAMA, Hamilton Anxiety Scale; HAMD, Hamilton depression scale, HS, healthy control; CS, concomitant strabismus. ^a^Two-sample t-test (two-tailed). ^b^Chi-square t-test*.

### Cortical Thickness Changes in CS Patients

**Figure 1:**
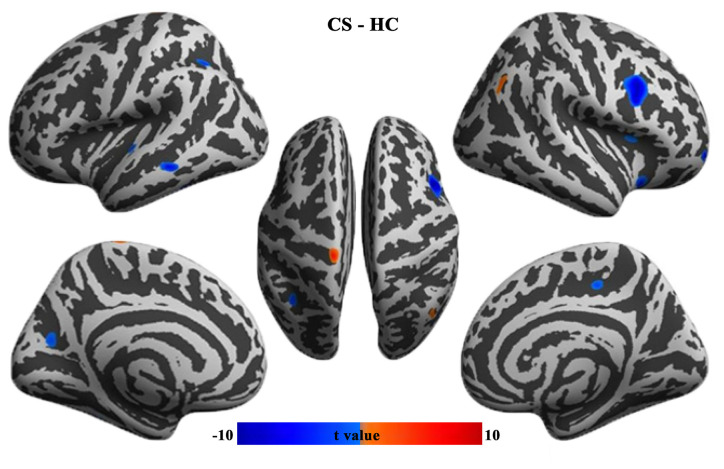
Group differences in cortical thickness between CS patients and HCs (FWE corrected, p < 0.05). CS, concomitant strabismus; HC, healthy control; FWE, family-wise error rate.

In contrast to HCs, patients with CS showed increased cortical thickness in the left precentral gyrus and right angular gyrus, while decreased cortical thickness in the left intraparietal sulcus, parieto-occipital sulcus, superior and middle temporal gyrus, right ventral premotor cortex, anterior insula, orbitofrontal cortex, and paracentral lobule (Figure 1).

### ALFF Changes in CS Patients

**Figure 2:**
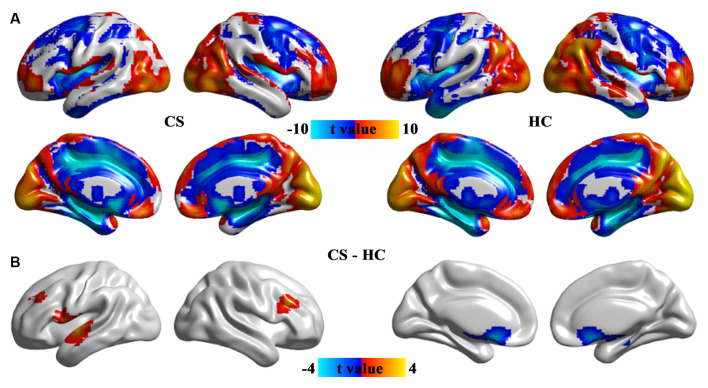
The pattern of ALFF in the CS and HC groups **(A)** and brain regions with significant group differences in ALFF **(B)**. Group differences in ALFF between the CS and HC groups were identified using a two-sample t-test. The statistical significance level was set at *p* < 0.05, GRF corrected. ALFF, the amplitude of low-frequency fluctuation; CS, concomitant strabismus; HC, healthy control.

Contrast to HCs, the patients with CS showed increased ALFF in the increased ALFF in the prefrontal cortex and superior temporal gyrus, and decreased ALFF in the caudate and hippocampus (Figure 2 and Table 2).

**Table 2:** Group differences in ALFF between CS patients and HCs.

	Brain regions	L/R	Peak MNI coordinates			Cluster size (voxels)	*t* value
			*x*	*y*	*z*		
CS > HC	Rolandic operculum	L	−60	9	3	209	3.82
	Middle frontal gyrus	L	−42	36	39	44	3.30
	Superior temporal gyrus	L	−51	−12	−3	209	4.44
	Inferior frontal gyrus	R	48	27	30	47	3.59
CS< HC	Caudate	L	−6	−15	−18	284	−3.19
	Caudate	R	3	9	−15	284	−3.76
	Hippocampus	R	18	−15	−18	284	−3.26

*ALFF, amplitude of low-frequency fluctuation*.

### Correlation Results

There were no significant correlations between altered cortical thickness, ALFF values, and disease duration, HAMA, HAMD, and MoCA scores.

## Discussion

This is the first study to investigate the change of structure and function of brain regions in children with CS. In contrast to HCs, CS patients showed increased cortical thickness in the precentral gyrus and angular gyrus, decreased cortical thickness in the frontal, parietal, occipital, and temporal cortex regions. Meanwhile, CS patients exhibited increased ALFF in the frontal and temporal cortex regions and decreased ALFF in the subcortical regions. These results provided novel evidence to deepen our understanding of the pathological mechanism underlying children with CS.

Children with CS showed abnormal cortical thickness in brain regions subserving visual information processing and eye movement. The occipitotemporal sulcus has been called a visual word form area (Mano et al., 2013), with the posterior part structurally connected to the intraparietal sulcus and involved in visual feature extraction; and the anterior part structurally connected to the angular gyrus and involved in integrating information with the language network (Wang et al., 2012; Wang et al., 2015b; Lerma-Usabiaga et al., 2018). Parieto-occipital sulcus acts as an interface between the dorsal and ventral streams of visual processing in disparity-defined near and far space processing (Wang et al., 2016, 2019). The precentral gyrus is a part of the primary motor cortex which is involved in ocular movement (Halsband et al., 1994). The frontal eye field (FEF) controls saccade modulation (Ohayon et al., 2013) and triggers the generation of saccade movements (Keller et al., 2008). The middle temporal gyrus is responsible for three-dimensional surface orientation and retinal image velocities (Nguyenkim and DeAngelis, 2003; Xu et al., 2015, 2019a). The lesions of these regions lead to ocular movement disorders. Significantly decreased GMV and WMV in the premotor cortex and middle temporal gyrus in adults with CS (Ouyang et al., 2017), and increased GMV in the FEF in adult strabismus (Chan et al., 2004) were previously reported. Strabismic amblyopia showed abnormal mean diffusion in the occipital tracts and the association tracts connecting the visual cortex to the frontal and temporal lobes (Duan et al., 2015). Moreover, strabismic amblyopia patients showed lower degree centrality value in the angular gyrus (Wu et al., 2020), and increased ReHo values in the cingulate gyrus (Huang et al., 2016b). Our results together with previous findings implied that CS may lead to the aberrant structure of visual cortex regions for impaired oculomotor in the CS patients.

More interestingly, CS patients reported decreased cortical thickness and increased ALFF in the high-order cognitive regions. PFC is interconnected with visual cortical areas and influences visual representations in sensory areas through attentional demands (Yeterian et al., 2012). The frontomarginal gyrus is a part of the orbitofrontal cortex which plays a central role in visuo-affective prediction (Chaumon et al., 2014). Lateral PFC modulates high-order attention control and provides a substrate for discriminating between perceptual and mnemonic representations of visual features (Wu et al., 2016; Mendoza-Halliday and Martinez-Trujillo, 2017). The superior temporal gyrus is involved in the comprehension of language (Kovelman et al., 2015; Wang et al., 2015a) and auditory-visual processing (Reale et al., 2007). Insula and rolandic operculum are involved in a multisensory integration center for spatial orientation and ocular motor function (Dieterich et al., 1998; Nagai et al., 2007; Wang et al., 2018, 2020). The abnormal structure and function of these high-order cognition regions were previously reported to be associated with strabismus. Abnormal fractional anisotropy and mean diffusivity values in the superior temporal gyrus and lateral PFC regions were reported in CS adults (Huang et al., 2016a). Moreover, increased degree centrality values in the superior temporal gyrus and decreased values in the lateral PFC in adults with comitant exotropia strabismus (Tan et al., 2018). Decreased long-range functional connectivity density in the insula, rolandic operculum, and lateral PFC were found in children with anisometropic amblyopia (Wang et al., 2014). Consistent with previous studies, our result further revealed the altered structure and function in high-order cortical regions in children with CS and highlighted the neural basis underlying abnormal top-down control visual attention in patients.

CS patients also exhibited decreased ALFF in the caudate and hippocampus. The caudate and hippocampus are a part of the visual loop, which is involved in eye movement control and visual working memory, in particular selecting or gating which visual representations should be maintained and processed in working memory (Seger, 2013). The caudate is implicated in dynamic visual information processing, saccade control, and eye movement (Nagypál et al., 2015), while the hippocampus is implicated in visual discrimination of complex spatial scene stimuli (Lee et al., 2012). A voxel-mirrored homotopic connectivity study reported increased interhemispheric functional connectivity in the caudate and hippocampus in patients with strabismic amblyopia (Zhang et al., 2021). In the present study, we speculated that the dysfunction of subcortical regions perhaps contributed to the dysfunction of eye movement and visual working memory in children with CS.

There are still some limitations in our study. First, this is a cross-study and longitudinal studies are warranted to better reveal the neuropathology of CS. Second, only cortical thickness was studied in this study. How cortical area and complexity changes in CS are deserved to be investigated since the three indices reflect different genetic information. Third, the sample size of this study is small. The expiations of these results should be cautious and the results need to be further validated in future studies.

## Conclusion

In this study, we reported abnormal structure and function in children with CS for the first time. CS patients exhibited not only abnormal oculomotor control but also impaired high-order cognitive and affective functions, which provides a neural basis for understanding the pathophysiology of CS. These findings suggest the importance of investigating the brain alteration to improve our understanding of CS. Future research is needed to further explore the neural basis of CS using the brain connectivity method.

## Data Availability Statement

The raw data supporting the conclusions of this article will be made available by the authors, without undue reservation.

## Ethics Statement

The studies involving human participants were reviewed and approved by affiliated Xi’an Central Hospital of Xi’an Jiaotong University. The patients/participants provided their written informed consent to participate in this study.

## Author Contributions

All the authors have made substantial contribution to this work. All authors contributed to the article and approved the submitted version.

## Conflict of Interest

The authors declare that the research was conducted in the absence of any commercial or financial relationships that could be construed as a potential conflict of interest.

## Publisher’s Note

All claims expressed in this article are solely those of the authors and do not necessarily represent those of their affiliated organizations, or those of the publisher, the editors and the reviewers. Any product that may be evaluated in this article, or claim that may be made by its manufacturer, is not guaranteed or endorsed by the publisher.

## Footnotes

^1^ http://rfmri.org/dpabi

^2^ http://www.fil.ion.ucl.ac.uk/spm/software/spm12/

## References

[B1] Archer S. M. Musch D. C. Wren P. A. Guire K. E. Del Monte M. A. (2005). Social and emotional impact of strabismus surgery on quality of life in children. J. Am. Assoc. Pediatric Ophthalmol. Strabismus 9, 148–151. 10.1016/j.jaapos.2004.12.006 15838442

[B2] Chan S.-T. Tang K.-W. Lam K.-C. Chan L.-K. Mendola J. D. Kwong K. K. (2004). Neuroanatomy of adult strabismus: a voxel-based morphometric analysis of magnetic resonance structural scans. Neuroimage 22, 986–994. 10.1016/j.neuroimage.2004.02.021 15193630

[B3] Chaumon M. Kveraga K. Barrett L. F. Bar M. (2014). Visual predictions in the orbitofrontal cortex rely on associative content. Cereb. Cortex 24, 2899–2907. 10.1093/cercor/bht146 23771980 PMC4193460

[B4] Dale A. M. Fischl B. Sereno M. I. (1999). . Cortical surface-based analysis: I. Segmentation and surface reconstruction. Neuroimage 9, 179–194. 10.1006/nimg.1998.0395 9931268

[B5] Dale A. M. Sereno M. I. (1993). Improved localizadon of cortical activity by combining EEG and MEG with MRI cortical surface reconstruction: a linear approach. J. Cogn. Neurosci. 5, 162–176. 10.1162/jocn.1993.5.2.162 23972151

[B6] Dieterich M. Bucher S. F. Seelos K. C. Brandt T. (1998). Horizontal or vertical optokinetic stimulation activates visual motion-sensitive, ocular motor and vestibular cortex areas with right hemispheric dominance. An fMRI study. Brain 121, 1479–1495. 10.1093/brain/121.8.1479 9712010

[B7] Duan Y. Norcia A. M. Yeatman J. D. Mezer A. (2015). The structural properties of major white matter tracts in strabismic amblyopia. Invest. Ophthalmol. Vis. Sci. 56, 5152–5160. 10.1167/iovs.15-17097 26241402 PMC4525637

[B8] Fischl B. Salat D. H. Busa E. Albert M. Dieterich M. Haselgrove C.. (2002). Whole brain segmentation: automated labeling of neuroanatomical structures in the human brain. Neuron 33, 341–355. 10.1016/s0896-6273(02)00569-x 11832223

[B9] Fischl B. Sereno M. I. Dale A. M. (1999). Cortical surface-based analysis - II: Inflation, flattening and a surface-based coordinate system. Neuroimage 9, 195–207. 10.1006/nimg.1998.0396 9931269

[B10] Halsband U. Matsuzaka Y. Tanji J. (1994). Neuronal activity in the primate supplementary, pre-supplementary and premotor cortex during externally and internally instructed sequential movements. Neurosci. Res. 20, 149–155. 10.1016/0168-0102(94)90032-9 7808697

[B11] Huang X. Li H.-J. Zhang Y. Peng D.-C. Hu P.-H. Zhong Y.-L.. (2016a). Microstructural changes of the whole brain in patients with comitant strabismus: evidence from a diffusion tensor imaging study. Neuropsychiatr. Dis. Treat. 12, 2007–2014. 10.2147/NDT.S108834 27574432 PMC4991538

[B12] Huang X. Li S.-H. Zhou F.-Q. Zhang Y. Zhong Y.-L. Cai F.-Q.. (2016b). Altered intrinsic regional brain spontaneous activity in patients with comitant strabismus: a resting-state functional MRI study. Neuropsychiatr. Dis. Treat. 12, 1303–1308. 10.2147/NDT.S105478 27350747 PMC4902244

[B13] Keller E. L. Lee B.-T. Lee K.-M. (2008). Frontal eye field signals that may trigger the brainstem saccade generator. Prog. Brain Res. 171, 107–114. 10.1016/S0079-6123(08)00614-6 18718288

[B14] Kovelman I. Wagley N. Hay J. S. Ugolini M. Bowyer S. M. Lajiness-O’Neill R.. (2015). Multimodal imaging of temporal processing in typical and atypical language development. Ann. N. Y. Acad. Sci. 1337, 7–15. 10.1111/nyas.12688 25773611

[B15] Lee A. C. Yeung L.-K. Barense M. D. (2012). The hippocampus and visual perception. Front. Hum. Neurosci. 6:91. 10.3389/fnhum.2012.00091 22529794 PMC3328126

[B16] Lerma-Usabiaga G. Carreiras M. Paz-Alonso P. M. (2018). Converging evidence for functional and structural segregation within the left ventral occipitotemporal cortex in reading. Proc. Natl. Acad. Sci. 115, E9981–E9990. 10.1073/pnas.1803003115 30224475 PMC6196482

[B17] Mano Q. R. Humphries C. Desai R. H. Seidenberg M. S. Osmon D. C. Stengel B. C.. (2013). The role of left occipitotemporal cortex in reading: reconciling stimulus, task and lexicality effects. Cereb. Cortex 23, 988–1001. 10.1093/cercor/bhs093 22505661 PMC3593581

[B18] Mendoza-Halliday D. Martinez-Trujillo J. C. (2017). Neuronal population coding of perceived and memorized visual features in the lateral prefrontal cortex. Nat. Commun. 8:15471. 10.1038/ncomms15471 28569756 PMC5461493

[B19] Nagai M. Kishi K. Kato S. (2007). Insular cortex and neuropsychiatric disorders: a review of recent literature. Eur. Psychiatry 22, 387–394. 10.1016/j.eurpsy.2007.02.006 17416488

[B20] Nagypál T. Gombkötho P. Barkóczi B. Benedek G. Nagy A. (2015). Activity of caudate nucleus neurons in a visual fixation paradigm in behaving cats. PLoS One 10:e0142526. 10.1371/journal.pone.0142526 26544604 PMC4636356

[B21] Nguyenkim J. D. DeAngelis G. C. (2003). Disparity-based coding of three-dimensional surface orientation by macaque middle temporal neurons. J. Neurosci. 23, 7117–7128. 10.1523/JNEUROSCI.23-18-07117.2003 12904472 PMC6740667

[B22] Ohayon S. Grimaldi P. Schweers N. Tsao D. Y. (2013). Saccade modulation by optical and electrical stimulation in the macaque frontal eye field. J. Neurosci. 33, 16684–16697. 10.1523/JNEUROSCI.2675-13.2013 24133271 PMC3797379

[B23] Ouyang J. Yang L. Huang X. Zhong Y.-L. Hu P.-H. Zhang Y.. (2017). The atrophy of white and gray matter volume in patients with comitant strabismus: Evidence from a voxel-based morphometry study. Mol. Med. Rep. 16, 3276–3282. 10.3892/mmr.2017.7006 28713925 PMC5547961

[B24] Oystreck D. T. Lyons C. J. (2012). Comitant strabismus: perspectives, present and future. Saudi J. Ophthalmol. 26, 265–270. 10.1016/j.sjopt.2012.05.002 23961004 PMC3729504

[B25] Pirnia T. Joshi S. H. Leaver A. M. Vasavada M. Njau S. Woods R. P.. (2016). Electroconvulsive therapy and structural neuroplasticity in neocortical, limbic and paralimbic cortex. Transl. Psychiatry 6:e832. 10.1038/tp.2016.102 27271858 PMC4931600

[B26] Reale R. Calvert G. Thesen T. Jenison R. Kawasaki H. Oya H.. (2007). Auditory-visual processing represented in the human superior temporal gyrus. Neuroscience 145, 162–184. 10.1016/j.neuroscience.2006.11.036 17241747

[B27] Robaei D. Rose K. A. Kifley A. Cosstick M. Ip J. M. Mitchell P. (2006). Factors associated with childhood strabismus: findings from a population-based study. Ophthalmology 113, 1146–1153. 10.1016/j.ophtha.2006.02.019 16675019

[B28] Seger C. A. (2013). The visual corticostriatal loop through the tail of the caudate: circuitry and function. Front. Syst. Neurosci. 7:104. 10.3389/fnsys.2013.00104 24367300 PMC3853932

[B29] Singh M. K. Kesler S. R. Hadi Hosseini S. M. Kelley R. G. Amatya D. Hamilton J. P.. (2013). Anomalous gray matter structural networks in major depressive disorder. Biol. Psychiatry 74, 777–785. 10.1016/j.biopsych.2013.03.005 23601854 PMC3805751

[B30] Tan G. Dan Z.-R. Zhang Y. Huang X. Zhong Y.-L. Ye L.-H.. (2018). Altered brain network centrality in patients with adult comitant exotropia strabismus: a resting-state fMRI study. Int. J. Med. Res. 46, 392–402. 10.1177/0300060517715340 28679330 PMC6011327

[B31] Tan G. Huang X. Zhang Y. Wu A.-H. Zhong Y.-L. Wu K.. (2016). A functional MRI study of altered spontaneous brain activity pattern in patients with congenital comitant strabismus using amplitude of low-frequency fluctuation. Neuropsychiatr. Dis. Treat. 12, 1243–1250. 10.2147/NDT.S104756 27284244 PMC4882152

[B32] Wang J. Becker B. Wang L. Li H. Zhao X. Jiang T. (2019). Corresponding anatomical and coactivation architecture of the human precuneus showing similar connectivity patterns with macaques. Neuroimage 200, 562–574. 10.1016/j.neuroimage.2019.07.001 31276799

[B33] Wang J. Fan L. Wang Y. Xu W. Jiang T. Fox P. T.. (2015a). Determination of the posterior boundary of wernicke’s area based on multimodal connectivity profiles. Hum. Brain Mapp. 36, 1908–1924. 10.1002/hbm.22745 25619891 PMC4782781

[B34] Wang J. Yang Y. Fan L. Xu J. Li C. Liu Y.. (2015b). Convergent functional architecture of the superior parietal lobule unraveled with multimodal neuroimaging approaches. Hum. Brain Mapp. 36, 238–257. 10.1002/hbm.22626 25181023 PMC4268275

[B35] Wang J. Fan L. Zhang Y. Liu Y. Jiang D. Zhang Y.. (2012). Tractography-based parcellation of the human left inferior parietal lobule. Neuroimage 63, 641–652. 10.1016/j.neuroimage.2012.07.045 22846658

[B36] Wang T. Li Q. Guo M. Peng Y. Li Q. Qin W.. (2014). Abnormal functional connectivity density in children with anisometropic amblyopia at resting-state. Brain Res. 1563, 41–51. 10.1016/j.brainres.2014.03.015 24661911

[B37] Wang A. Li Y. Zhang M. Chen Q. (2016). The role of parieto-occipital junction in the interaction between dorsal and ventral streams in disparity-defined near and far space processing. PLoS One 11:e0151838. 10.1371/journal.pone.0151838 26999674 PMC4801215

[B38] Wang J. Wei Q. Bai T. Zhou X. Sun H. Becker B.. (2017). Electroconvulsive therapy selectively enhanced feedforward connectivity from fusiform face area to amygdala in major depressive disorder. Soc. Cogn .Affect Neurosci. 12, 1983–1992. 10.1093/scan/nsx100 28981882 PMC5716231

[B39] Wang L. Wei Q. Wang C. Xu J. Wang K. Tian Y.. (2020). Altered functional connectivity patterns of insular subregions in major depressive disorder after electroconvulsive therapy. Brain Imaging Behav. 14, 753–761. 10.1007/s11682-018-0013-z 30610527

[B40] Wang C. Wu H. Chen F. Xu J. Li H. Li H.. (2018). Disrupted functional connectivity patterns of the insula subregions in drug-free major depressive disorder. J. Affect. Disord. 234, 297–304. 10.1016/j.jad.2017.12.033 29587165

[B41] Wu Y. Wang J. Zhang Y. Zheng D. Zhang J. Rong M.. (2016). The neuroanatomical basis for posterior superior parietal lobule control lateralization of visuospatial attention. Front. Neuroanat. 10:32. 10.3389/fnana.2016.00032 27047351 PMC4805595

[B42] Wu K.-R. Yu Y.-J. Tang L.-Y. Chen S.-Y. Zhang M.-Y. Sun T.. (2020). Altered brain network centrality in patients with adult strabismus with amblyopia: a resting-state functional magnetic resonance imaging (fMRI) study. Med. Sci. Monit. 26:e925856. 10.12659/MSM.925856 33226973 PMC7693780

[B43] Xu J. Lyu H. Li T. Xu Z. Fu X. Jia F.. (2019a). Delineating functional segregations of the human middle temporal gyrus with resting-state functional connectivity and coactivation patterns. Hum. Brain Mapp. 40, 5159–5171. 10.1002/hbm.24763 31423713 PMC6865466

[B44] Xu J. Wang J. Bai T. Zhang X. Li T. Hu Q.. (2019b). Electroconvulsive therapy induces cortical morphological alterations in major depressive disorder revealed with surface-based morphometry analysis. Int. J. Neural Syst. 29:1950005. 10.1142/S0129065719500059 31387489

[B45] Xu J. Wang J. Fan L. Li H. Zhang W. Hu Q.. (2015). Tractography-based parcellation of the human middle temporal gyrus. Sci. Rep. 5:18883. 10.1038/srep18883 26689815 PMC4686935

[B46] Yan X. Wang Y. Xu L. Liu Y. Song S. Ding K.. (2019). Altered functional connectivity of the primary visual cortex in adult comitant strabismus: a resting-state functional MRI study. Curr. Eye Res. 44, 316–323. 10.1080/02713683.2018.1540642 30375900

[B47] Yeterian E. H. Pandya D. N. Tomaiuolo F. Petrides M. (2012). The cortical connectivity of the prefrontal cortex in the monkey brain. Cortex 48, 58–81. 10.1016/j.cortex.2011.03.004 21481342 PMC3161133

[B48] Zhang S. Gao G.-P. Shi W.-Q. Li B. Lin Q. Shu H.-Y.. (2021). Abnormal interhemispheric functional connectivity in patients with strabismic amblyopia: a resting-state fMRI study using voxel-mirrored homotopic connectivity. BMC Ophthalmol. 21:255. 10.1186/s12886-021-02015-0 34107904 PMC8188699

